# Calcium/calmodulin‐dependent kinase 2 mediates Epac‐induced spontaneous transient outward currents in rat vascular smooth muscle

**DOI:** 10.1113/JP274754

**Published:** 2017-08-14

**Authors:** Edward S. A. Humphries, Tomoko Kamishima, John M. Quayle, Caroline Dart

**Affiliations:** ^1^ Institutes of Integrative Biology; ^2^ Translational Medicine University of Liverpool Liverpool UK

**Keywords:** Epac, CaMKII, vascular smooth muscle

## Abstract

**Key points:**

- The Ca^2+^ and redox‐sensing enzyme Ca^2+^/calmodulin‐dependent kinase 2 (CaMKII) is a crucial and well‐established signalling molecule in the heart and brain.
- In vascular smooth muscle, which controls blood flow by contracting and relaxing in response to complex Ca^2+^ signals and oxidative stress, surprisingly little is known about the role of CaMKII.
- The vasodilator‐induced second messenger cAMP can relax vascular smooth muscle via its effector, exchange protein directly activated by cAMP (Epac), by activating spontaneous transient outward currents (STOCs) that hyperpolarize the cell membrane and reduce voltage‐dependent Ca^2+^ influx. How Epac activates STOCs is unknown.
- In the present study, we map the pathway by which Epac increases STOC activity in contractile vascular smooth muscle and show that a critical step is the activation of CaMKII.
- To our knowledge, this is the first report of CaMKII activation triggering cellular activity known to induce vasorelaxation.

**Abstract:**

Activation of the major cAMP effector, exchange protein directly activated by cAMP (Epac), induces vascular smooth muscle relaxation by increasing the activity of ryanodine (RyR)‐sensitive release channels on the peripheral sarcoplasmic reticulum. Resultant Ca^2+^ sparks activate plasma membrane Ca^2+^‐activated K^+^ (BK_Ca_) channels, evoking spontaneous transient outward currents (STOCs) that hyperpolarize the cell and reduce voltage‐dependent Ca^2+^ entry. In the present study, we investigate the mechanism by which Epac increases STOC activity. We show that the selective Epac activator 8‐(4‐chloro‐phenylthio)‐2′‐*O*‐methyladenosine‐3′, 5‐cyclic monophosphate‐AM (8‐pCPT‐AM) induces autophosphorylation (activation) of calcium/calmodulin‐dependent kinase 2 (CaMKII) and also that inhibition of CaMKII abolishes 8‐pCPT‐AM‐induced increases in STOC activity. Epac‐induced CaMKII activation is probably initiated by inositol 1,4,5‐trisphosphate (IP_3_)‐mobilized Ca^2+^: 8‐pCPT‐AM fails to induce CaMKII activation following intracellular Ca^2+^ store depletion and inhibition of IP_3_ receptors blocks both 8‐pCPT‐AM‐mediated CaMKII phosphorylation and STOC activity. 8‐pCPT‐AM does not directly activate BK_Ca_ channels, but STOCs cannot be generated by 8‐pCPT‐AM in the presence of ryanodine. Furthermore, exposure to 8‐pCPT‐AM significantly slows the initial rate of [Ca^2+^]_i_ rise induced by the RyR activator caffeine without significantly affecting the caffeine‐induced Ca^2+^ transient amplitude, a measure of Ca^2+^ store content. We conclude that Epac‐mediated STOC activity (i) occurs via activation of CaMKII and (ii) is driven by changes in the underlying behaviour of RyR channels. To our knowledge, this is the first report of CaMKII initiating cellular activity linked to vasorelaxation and suggests novel roles for this Ca^2+^ and redox‐sensing enzyme in the regulation of vascular tone and blood flow.

## Key points

- The Ca^2+^ and redox‐sensing enzyme Ca^2+^/calmodulin‐dependent kinase 2 (CaMKII) is a crucial and well‐established signalling molecule in the heart and brain.
- In vascular smooth muscle, which controls blood flow by contracting and relaxing in response to complex Ca^2+^ signals and oxidative stress, surprisingly little is known about the role of CaMKII.
- The vasodilator‐induced second messenger cAMP can relax vascular smooth muscle via its effector, exchange protein directly activated by cAMP (Epac), by activating spontaneous transient outward currents (STOCs) that hyperpolarize the cell membrane and reduce voltage‐dependent Ca^2+^ influx. How Epac activates STOCs is unknown.
- In the present study, we map the pathway by which Epac increases STOC activity in contractile vascular smooth muscle and show that a critical step is the activation of CaMKII.
- To our knowledge, this is the first report of CaMKII activation triggering cellular activity known to induce vasorelaxation.

**Abbreviations**
2‐APB: 2‐aminoethoxydiphenyl borate
8‐pCPT‐AM: 8‐(4‐chloro‐phenylthio)‐2′‐*O*‐methyladenosine‐3′, 5‐cyclic monophosphate‐AM
AIP: autocamtide‐2‐inhibitory peptide
BK_Ca_: large‐conductance Ca^2+^‐activated potassium channel
CaMKII: Ca^2+^/calmodulin kinase II
Epac: exchange protein directly activated by cAMP
Ca_V_1.2: voltage‐dependent L‐type Ca^2+^ channel
GEF: guanine nucleotide exchange factor
GST: glutathione *S*‐transferase
IP_3_: inositol 1,4,5‐trisphosphate
IP_3_R: inositol 1,4,5‐trisphosphate receptor
PKA: cAMP‐dependent protein kinase
PLC: phospholipase C
RalGDS‐RBD: Ral‐guanine nucleotide‐dissociation stimulator Rap‐binding domain
RMASMC: rat mesenteric arterial smooth muscle cell
RIPA: radioimmunoprecipitation assay
RyR: ryanodine receptors
SDS: sodium dodecyl sulfate
SERCA: sarcoplasmic reticulum Ca^2+^ ATPase
SR: sarcoplasmic reticulum
STOCs: spontaneous transient outward currents

## Introduction

The opening of plasma membrane large‐conductance Ca^2+^‐activated potassium (BK_Ca_) channels in vascular smooth muscle cells is triggered by localized Ca^2+^ release from the subjacent sarcoplasmic reticulum (SR) (Jaggar *et al*. 2000). The resultant outward K^+^ currents, spontaneous transient outward currents (STOCs), hyperpolarize the cell membrane and decrease Ca^2+^ entry via voltage‐dependent L‐type (Ca_v_1.2) Ca^2+^ channels. Ca_v_1.2 channels provide the main Ca^2+^ influx pathway in vascular smooth muscle and, consequently, membrane hyperpolarization lowers global intracellular Ca^2+^ and induces muscle relaxation (Moosmang *et al*. 2003). The coupling between localized SR Ca^2+^ release (termed Ca^2+^ sparks) and the generation of STOCs is thus a central mechanism that opposes arterial constriction induced by intravascular pressure or vasoactive transmitters. Indeed, inhibition of Ca^2+^ spark activity elicits contraction in most vascular beds, emphasizing the functional significance of this negative‐feedback pathway (Jaggar *et al*. 2000). Ca^2+^ sparks themselves originate from the opening of single or clustered groups of ryanodine‐sensitive Ca^2+^ release channels (RyRs) on the peripheral SR. RyRs respond to changes in both cytosolic and luminal SR Ca^2+^ levels (ZhuGe *et al*. 1999) and sparks can occur spontaneously or be triggered directly or indirectly by the influx of extracellular Ca^2+^ (Earley *et al*. 2005; Essin *et al*. 2007).

The ability of Ca^2+^ sparks to affect the membrane potential through the activation of cell‐surface BK_Ca_ channels means that changes of spark frequency and/or amplitude influences vascular tone. In arterial tissue and isolated vascular myocytes, nitric oxide and a range of β‐adrenergic agonists induce vasorelaxation by increasing both spark frequency and the activity of BK_Ca_ channels (Jaggar *et al*. 1998; Porter *et al*. 1998; Mauban *et al*. 2001; Pucovsky *et al*. 2002; Kim *et al*. 2006). There is also strong evidence that the coupling between spark and STOC activity regulates vascular resistance and blood pressure *in vivo*. In mice, disruption of the gene encoding the regulatory β‐subunit of BK_Ca_ channels leads to defective coupling between sparks and STOCs and is associated with elevated blood pressure and left ventricular hypertrophy (Brenner *et al*. 2000; Pluger *et al*. 2000). Hypertensive rat models have decreased expression of BK_Ca_ β‐subunits and BK_Ca_ channels with low Ca^2+^ sensitivity that fail to respond normally to Ca^2+^ sparks (Amberg & Santana, 2003). In humans, population‐based genetic studies have identified a single‐nucleotide gain‐of‐function substitution in the β_1_ subunit gene that results in BK_Ca_ channels with a heightened sensitivity to Ca^2+^. This mutation is associated with low prevalence of diastolic hypertension and has a strong protective effect against myocardial infarction and stroke (Fernandez‐Fernandez *et al*. 2004; Senti *et al*. 2005). Enhanced expression of the pore‐forming α subunits of BK_Ca_ channel is also widely reported in hypertensive animal models, suggesting that these channels may form part of a compensatory mechanism in response to abnormal vascular tone (Cox & Rusch, 2002).

Mechanisms by which vasorelaxants increase spark‐STOC activity have not been fully defined. β‐adrenergic agonists that stimulate this pathway bind to cell surface G_s_‐coupled receptors that elevate intracellular levels of cAMP, indicating a possible role for this second messenger. Downstream of cAMP lies the major cAMP effector, exchange protein directly activated by cAMP (Epac). Epacs are guanine nucleotide exchange factors (GEFs) for the small Ras‐related G proteins Rap1 and Rap2 (de Rooij *et al*. 1998; Kawasaki *et al*. 1998) and are abundant in the vasculature where they modulate cytokine signalling, strengthen endothelial barrier function and participate in vascular remodelling (Roberts & Dart, 2014). Recent studies suggest additional roles for Epac as a potent vasodilator (Sukhanova *et al*. 2006; Roscioni *et al*. 2011; Zieba *et al*. 2011; Roberts *et al*. 2013; Stott *et al*. 2016). β‐adrenergic and prostacyclin‐induced elevation of cAMP changes the sensitivity of the contractile proteins through Rap‐initiated alteration of myosin light chain phosphorylation (Sukhanova *et al*. 2006; Roscioni *et al*. 2011; Zieba *et al*. 2011) and, using the Epac‐specific cAMP homologue 8‐(4‐chloro‐phenylthio)‐2′‐*O*‐methyladenosine‐3′,5‐cyclic monophosphate‐AM (8‐pCPT‐AM), we have recently shown that Epac increases Ca^2+^ spark and STOC activity to induce membrane hyperpolarization and relaxation of vascular smooth muscle (Roberts *et al*. 2013). Importantly, these effects persist in the presence of potent and selective cAMP‐dependent kinase (PKA) inhibitors and suggest an important vasorelaxant pathway that is distinct from the more traditional cAMP‐PKA axis.

The mechanism by which Epac activation increases STOC activity in smooth muscle cells is unknown. In cardiomyocytes, Epac activation increases Ca^2+^ release through Ca^2+^/calmodulin‐dependent kinase 2 (CaMKII)‐dependent phosphorylation of both RyRs and phospholamban, a small pentameric protein complex that controls the activity of the SR Ca^2+^ ATPase (SERCA) (Oestreich *et al*. 2009). Phosphorylation by CaMKII increases cardiac RyR2 activity by enhancing its sensitivity to Ca^2+^ (Wehrens *et al*. 2004), whereas CaMKII‐induced phosphorylation of phospholamban relieves its inhibition of SERCA, thereby increasing Ca^2+^ uptake into stores and thus store content and RyR activity. Although CaMKII is recognized as an important signalling molecule in the heart, surprisingly little is known about its role in controlling vascular tone. In the present study, we assess the possible involvement of CaMKII in Epac‐mediated increases in STOC activity in contractile vascular smooth muscle cells.

## Methods

### Ethical approval

Tissues were obtained from adult male Wistar rats (175–225 g; Charles River Laboratories, Wilmington, MA, USA) and from wild‐type and CaMKIIδ and CaMKIIγ knockout mice (C57B/6 background; provided by Dr Tim Curtis, Queens University, Belfast, UK). Wild‐type mice were littermates of the homozygous knockouts. All animals were killed by a rising concentration of CO_2_ followed by cervical dislocation. The care and killing of animals conformed with the requirements of the UK Animals (Scientific Procedures) Act 1986 and was approved by the University of Liverpool and Queens University, Belfast local ethics committees.

#### Chemicals

The chemicals employed in the present study were KN‐93, NS11021 (Tocris Bioscience, St Louis, MO, USA), 8‐(4‐chlorophenylthio)‐2′‐*O*‐methyladenosine‐3′,5′‐cyclic monophosphate‐AM (8‐pCPT‐2′‐*O*‐Me‐cAMP‐AM; Biolog Life Science Institute, Bremen, Germany), Fura‐2‐pentapotassium (Thermo Fisher Scientific Inc., Waltham, MA, USA). All other chemicals were purchased from Sigma‐Aldrich (St Louis, MO, USA).

### Cell isolation

Rat mesenteric arterial smooth muscle cells (RMASMCs) were isolated by enzymatic digestion of first‐order branches of the rat superior mesenteric artery as described previously (Hayabuchi *et al*. 2001). Briefly, proximal first‐order branches of rat superior mesenteric arteries were dissected and placed in low Ca^2+^ buffer containing (mm): 134 NaCl, 6 KCl, 0.42 Na_2_HPO_4_, 0.44 NaH_2_PO_4_, 10 Hepes, 10 glucose, 1 MgCl_2_ and 0.1 CaCl_2_ (pH 7.4) at 35°C for 10 min. After incubation, branches were moved to the first stage digestion buffer consisting of low Ca^2+^ buffer containing papain (1.4 mg ml^−1^), 4‐dithioerythritol (0.9 mg ml^−1^) and bovine serum albumin 0.9 mg ml^−1^ for 8–10 min at 35°C. Branches were then washed three times in pre‐warmed low Ca^2+^ buffer and transferred to second stage digestion buffer consisting of low Ca^2+^ buffer containing hyaluronidase (0.9 mg ml^−1^), collagenase (1.4 mg ml^−1^) and bovine serum albumin (0.9 mg ml^−1^) at 35°C. The incubation period for second stage digestion varied from between 8 and 12 min. Branches were subsequently transferred to fresh low Ca^2+^ buffer and carefully washed before dispersion of cells by gentle trituration of the tissue through a heat‐polished glass pipette. RMASMCs were stored in low Ca^2+^ buffer on ice and used in experiments between 1 and 8 h after digestion.

### Electrophysiology

STOCs were recorded from single freshly isolated RMASMCs using the whole‐cell configuration with an Axopatch 200B amplifier (Molecular Devices, Sunnyvale, CA, USA) as described previously (Roberts *et al*. 2013). Currents were filtered at 5 kHz and digitized at 10 kHz using a Digidata 1320A interface (Molecular Devices). The pipette‐filling solution contained (mm): 140 KCl, 3 MgCl_2_, 0.2 EGTA, 10 Hepes and 3 Na_2_ATP (pH 7.2). Extracellular recording solutions contained (mm): 6 KCl, 134 NaCl, 1 MgCl_2_, 2 CaCl_2_ and 10 Hepes (pH 7.4). STOCs were recorded at a holding potential of −20 mV and defined as events deviating from the baseline by a factor of 4 standard deviations above baseline noise. Autoinhibitory‐2‐related peptide (1 μm) was placed in the intracellular solution and allowed to dialyse into the cell for 5 min before the start of recording. In experiments to assess direct effects of 8‐pCPT‐AM on BK_Ca_ channel activity, the pipette‐filling solution contained 300 nm free calcium as calculated by Maxchelator Ca‐Mg‐ATP‐EGTA Calculator, version 1.0, using constants from NIST database #46, version 8 (http://maxchelator.stanford.edu).

### Calcium fluorometry: measurement of SR Ca^2+^ content

Transient Ca^2+^ release from the SR was triggered by pulse applications of the RyR activator caffeine in single freshly isolated voltage clamped and fura‐2‐dialysed RMASMCs as described previously (Kamishima & Quayle, 2002). Cells were suspended in low Ca^2+^ extracellular solution containing (in mm) 145 NaCl, 6 KCl, 1 MgCl_2_, 0.1 CaCl_2_, 10 Hepes and 10 glucose (pH 7.4) and placed in a perspex perfusion chamber. Background fluorescence counts for dual excitation wavelengths were taken after establishing a tight seal but before achieving the whole‐cell clamp configuration. On achieving the whole‐cell configuration, the cell was dialysed with pipette‐filling solution containing (in mm) 145 CsCl, 3 MgCl_2_, 3 Na_2_ATP, 10 Hepes and 0.05 fura‐2 pentapotassium (pH 7.2 with CsOH). The cell membrane potential was held at −60 mV, and normal Ca^2+^ extracellular solution, where CaCl_2_ was raised to 3 mm, was superfused. The cell was illuminated alternately with 340 and 380 nm light (bandpass 5 nm) using a PTI DeltaRAM Illuminator (Horiba Scientific, Burlington, ON, Canada) and the emission signal was detected at 510 nm (40 nm bandpass). Temporal changes in photon counts were measured, backgrounds subtracted, and the complete ratio obtained at 50 Hz. Fluorescence signals and cell membrane potentials were acquired using FelixGX (Horiba Scientific). RyRs were activated by rapid application of 5 mm caffeine using a U tube superfusion system (Evans & Kennedy, 1994). After the third or fourth caffeine application, the extracellular solution was switched to normal Ca^2+^ solution containing either vehicle control (DMSO) or 8‐pCPT‐AM (5 μm), thereafter caffeine transients were continuously triggered at regular intervals. To convert the fluorescence ratio to [Ca^2+^]_i_, *in vitro* calibration was carried out using a range of EGTA‐buffered intracellular solutions containing a desired free Ca^2+^ calculated with MAXchelator. Fluorescence ratios in the absence of added CaCl_2_ (*R*_min_) and in the presence of saturating Ca^2+^ (*R*_max_) were decreased by 15% to account for viscosity (Poenie *et al*. 1986), and the *K*_d_ of fura‐2 was determined as 160 nm. To profile Ca^2+^ transients, three parameters, amplitude, duration and rate of cytosolic Ca^2+^ increase, were examined. Amplitude was measured as the [Ca^2+^]_i_ difference between resting and peak [Ca^2+^]_i_. Duration was determined as the time that [Ca^2+^]_i_ remained above the sum of resting [Ca^2+^]_i_ and a half‐amplitude. To examine the early stage of the cytosolic Ca^2+^ rise, data points from baseline to [Ca^2+^]_i_ of 200 nm were fit with a third‐order polynomial fit using Sigmaplot, version 13 (Systat Software Inc., Chicago, IL, USA) and the rate of Ca^2+^ increase was determined as a half‐time taken to reach a [Ca^2+^]_i_ of 200 nm. For each parameter, the value of the third Ca^2+^ transient after switching to the test solution was normalized against that of the Ca^2+^ transient immediately before solution change.

### SDS‐PAGE and immunoblotting

First‐ or second‐order branches of rat superior mesenteric arteries (five to eight branches) were homogenized in a pre‐cooled hand‐held homogenizer containing 100–500 μl of ice‐cold lysis buffer comprising (mm): 20 Tris‐HCl; 250 NaCl, 3 EDTA, 3 EGTA, 0.5% (v/v) Triton‐X 100 (pH 7.6), 1% (v/v) protease inhibitor cocktail (Sigma‐Aldrich) and 1X PhosSTOP Phosphatase Inhibitor Cocktail Tablets (Roche, Basel, Switzerland). Resultant lysates were placed on ice for 10 min then centrifuged at 16 100 *g* for 10 min at 4°C. Supernatants were removed and mixed 1:3 (v/v) with 4 × SDS sample buffer, before heating to 98°C for 10 min. Lysates for phospholamban immunoblots were prepared by lysis in either radioimmunoprecipitation assay (RIPA) buffer or 1 × SDS sample buffer to maximize phosphorylation preservation and increase protein solubilization. RIPA lysates were then mixed 1:3 with 4 × sample buffer as with Triton X‐100 based lysates. Samples were kept at −20°C until use. Proteins within the lysates were resolved by SDS‐PAGE on either 5%, 10% or 15% polyacrylamide‐Tris‐glycine gels (dependent on the protein of interest) and transferred electrophoretically onto nitrocellulose membranes (Hybond ECL; GE Healthcare, Little Chalfont, UK). When immunoblotting for phospholamban, proteins were transferred to 0.22 μm polyvinylidene fluoride membrane. Immunoblotting was performed as described previously (Sampson *et al*. 2003). Where indicated, membranes were stripped at room temperature for 1 h in 50 mm Tris‐HCl (pH 6.8), 1% SDS.

### Antibodies

For immunoblotting, the primary antibodies used were: phospho‐CaMKII (T^286^: dilution 1:1000); phospholamban (dilution 1:500), Rap 1A/B (dilution 1:1000) were from Cell Signaling Technology (Beverly, MA, USA). CaMKII (pan: dilution 1:2500) was from Abcam (Cambridge, MA, USA). Smooth muscle α actin (dilution 1:10,000) was from Sigma‐Aldrich. The secondary antibodies used were anti‐mouse IgG (H+L) HRP conjugated polyclonal antibody and anti‐rabbit IgG (H+L) HRP conjugated polyclonal antibody (Stratech Scientific Ltd, Newmarket, UK).

### Expression of GST‐RalGDS‐RBD and Rap1‐GTP pull‐down

GST‐RalGDS‐RBD fusion protein, consisting of glutathione *S*‐transferase (GST) fused to the Rap binding domain (RBD) of Ral guanine nucleotide dissociation stimulator (RalGDS), was expressed from the pGEX4T3‐GST‐RalGDS‐RBD plasmid, kindly donated by Professor Johannes Bos (University Medical Centre, Utrecht, The Netherlands), in the BL21 strain of *Escherichia coli* as described previously (Van Triest *et al*. 2001). Pull‐down of active (GTP‐bound) Rap was carried out as described previously (Roberts *et al*. 2013).

### Total RNA extraction and reverse transcription

Total RNA was extracted and purified from first‐ or second‐order branches of rat superior mesenteric arteries using an RNeasy Mini Kit® (Qiagen, Valancia, CA, USA) in accordance with the manufacturer's instructions. Extracted RNA was treated with DNase I by incubating 8 μl of total RNA, 1 μl of 10 × DNase I buffer and 1 μl of DNase I (1 U μl^−1^; Invitrogen, Carlsbad, CA, USA) at room temperature for 15 min. Then, 1 μl of EDTA (25 mmol l^−1^) was added and the reaction heated at 65°C for 10 min. First‐strand cDNA was synthesized using SuperScript® III reverse transcriptase (Invitrogen) in accordance with the manufacturer's instructions. The purity and concentration of the resulting cDNA template was determined by measuring absorbance at 260 and 280 nm using a NanoDrop spectrophotometer (Thermo Fisher Scientific Inc.).

### PCR

Touchdown PCR was carried out using HotStarTaq Master Mix Plus (Qiagen) in accordance with the manufacturer's instructions. Primers used to amplify specific CaMKII isoforms were: δ: Forward (5′‐GCATGTGGCGTCATCCTCTA‐3′) Reverse (5′‐TCCTTTACCCCATCCGGTT‐3′); γ: Forward (5′‐CATCCACCAGCATGACATCG‐3′) Reverse (5′‐CTTTCCTCAAGACCTCAGG‐3′). δ splice variants: Forward (5′‐CGGAAATTGAAGGGTGCCATC‐3′) Reverse (5′‐CCCTACCAGGTGTACGTGAG‐3′); γ splice variants: Forward (5′‐GAAGGGTGCCATCCTCACAA‐3′) Reverse (5′‐GTTACCAAGGGCTTCTGGCT‐3′). Following an initial 5 min denaturing step at 95°C, each reaction went through a touchdown PCR protocol for 15 cycles: 94°C for 1 min, annealing temperature (ranging from 65°C to 50°C, decreasing by 1°C each cycle) for 30 s, extension at 72°C for 1 min. This was followed by 25 cycles at the lowest annealing temperature. Final extension was at 72°C for 5 min. Products were analysed by running on a 3% agarose gel containing Midori Green (dilution 1:10,000; GC Biotech, Alphen aan den Rijn, The Netherlands) for ∼1 h at 80 V. Bands were excised under ultraviolet light and products purified using a QIAquick Gel Extraction kit (Qiagen) in accordance with the manufacturer's instructions. Products were verified by sequencing (GATC Biotech, Konstanz, Germany).

### Statistical analysis

Results are expressed as the mean ± SEM. Intergroup differences were analysed using repeated measures ANOVA followed by Newman–Keuls *post hoc* test, or Student's *t* test for simple comparisons; levels of significance were *P* < 0.05 (^*^) and *P* < 0.01 (^**^). For electrophysiological and calcium fluorometry recordings, *n* is the number of cells recorded from. Cells were from at least three different animals. For immunoblots, *n* is the number of experimental repeats. These repeats were on different days and with tissue taken from different animals.

## Results

### Selective Epac activation causes autophosphorylation of specific CaMKII isoforms in rat mesenteric artery

**Figure 1:**
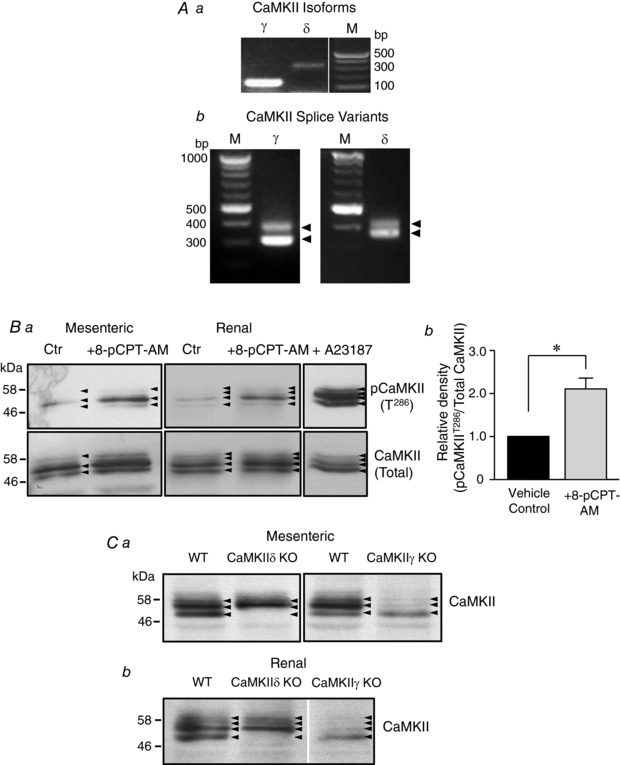
Selective activation of Epac causes autophosphorylation of specific CaMKII isoforms in rat mesenteric artery *A*, primers designed to amplify γ and δ isoforms (*Aa*) and γ and δ CaMKII splice variants (*Ab*) were used to probe rat mesenteric artery cDNA. PCR products were separated on a 3% agarose gel. M indicates DNA markers. *Ba*, Incubation of first‐order branches of rat mesenteric artery (left) or renal artery (right) with 8‐pCPT‐AM (10 μm) induces phosphorylation of specific CaMKII isoforms at Thr^286/7^ (pCaMKII (T^286/7^); upper). Arteries were incubated with vehicle control (DMSO) or 8‐pCPT‐AM (10 μm) for 5 min prior to homogenization. As a positive control, arteries were incubated in the Ca^2+^ ionophore, A23187, (12.5 μm) for 15 min prior to homogenization. Proteins within the arterial homogenates were separated on 10% polyacrylamide‐Tris gels and immunoblotted with an antibody directed against phospho‐CaMKII Thr^286/7^. The membrane was then stripped and re‐blotted with pan‐specific CaMKII antibodies (total CaMKII; lower; blots shown representative of three similar experiments). Film exposure time for all blots ∼5 min (*Bb*) Densitometry analysis of three similar blots. *C*, immunoblots of lysates from wild‐type mice mesenteric (*Ca*) or renal artery (*Cb*) using CaMKII antibodies showed a pattern of immunoreactive bands (arrowheads) similar to that of rat arterial lysates. In arterial lysates obtained from CaMKIIδ knockout animals, the lowest molecular weight band was absent. By contrast, in CaMKIIγ knockout lysates, the lower band remained but the higher molecular weight bands were missing. Film exposure time for all blots ∼10 min.

CaMKII is encoded by four closely related genes (α, β, γ and δ) with alternative splicing producing multiple variants (Soderling & Stull, 2001). The enzyme itself is a large complex made up of 12 subunits, with the predominant CaMKII holoenzymes found in vascular smooth muscle forming as a mixture of splice products from γ and δ genes (Singer, 2012). Reverse transcription‐PCR using cDNA derived from rat mesenteric artery as a template and primers designed to detect (i) CaMKII isoforms and (ii) γ and δ splice variants confirmed the presence of mRNA for at least two γ variants and two δ variants in this tissue (Fig. 1 *A*). Sequencing of these products revealed them to be γ predicted isoforms X18 and X16, and δ isoforms 2 and/or 3. Consistent with the expression of multiple CaMKII isoforms, immunoblots of arterial lysates using pan‐specific CaMKII antibodies produced three/four distinct immunoreactive bands (Fig. 1 *Ba*, lower). To assess the effect of Epac activation on CaMKII activity, we incubated arteries in the selective Epac activator 8‐pCPT‐2′‐*O*‐Me‐cAMP‐AM (8‐pCPT‐AM; 10 μm) or vehicle control (DMSO). As a positive control, we also incubated arteries in the Ca^2+^ ionophore A23187 (12.5 μm), which would be expected to globally raise intracellular Ca^2+^ and activate CaMKII. Immunoblot analysis of 8‐pCPT‐AM‐treated arterial lysates indicated phosphorylation of CaMKII at Thr286/7, an autophosphorylation site that indicates CaMKII activation, following 8‐pCPT‐AM exposure (*n* = 3) (Fig. 1 *Ba*, upper, *Bb*). Of the three/four immunoreactive bands detected by pan‐specific anti‐CaMKII, one band (second from bottom) strongly bound phospho‐CaMKII antibodies against Thr286/7 following 8‐pCPT‐AM treatment. Exposure to A23187 induced phosphorylation of all immunoreactive bands detected by pan‐specific anti‐CaMKII (Fig. 1 *Ba*, right).

To identify the CaMKII isoform/s differentially phosphorylated by Epac activation, we used immunoblotting to analyse arterial tissue obtained from mice in which the gene encoding either CaMKIIδ or CaMKIIγ had been globally knocked out (Backs *et al*. 2009; Backs *et al*. 2010). Immunoblots of arterial lysates from wild‐type mice using CaMKII antibodies showed a similar pattern of three/four immunoreactive bands as observed in rat tissue (Fig. 1 *Ca* and *Cb*). In arterial lysates obtained from CaMKIIδ knockout animals, the lowest molecular weight band was absent. By contrast, in CaMKIIγ knockout lysates, the lower band remained but the higher molecular weight bands were missing (Fig. 1 *Ca* and *Cb*). This suggests that Epac activation predominantly induces phosphorylation of CaMKII γ variants in vascular tissue. Whole arterial lysates contain a combination of different cell types and we thus undertook electrophysiological experiments on single isolated mesenteric myocytes to directly assess the effects of Epac‐mediated CaMKII activation in vascular smooth muscle.

### Inhibition of CaMKII attenuates the increase in STOC frequency mediated by Epac in RMASMCs

Selective activation of Epac with 8‐pCPT‐AM induces vasorelaxation by increasing both the frequency and amplitude of STOCs in rat mesenteric beds (Roberts *et al*. 2013). These 8‐pCPT‐AM‐mediated changes in STOC activity persist in the presence of the potent PKA inhibitor PKI‐(Myr‐14‐22)‐amide, indicating that they occur independently of PKA activation (Roberts *et al*. 2013). To assess whether 8‐pCPT‐induced activation of CaMKII is involved in the increase of STOC activity, we used the whole‐cell recording technique to record STOCs in mesenteric myocytes following activation of Epac in the presence and absence of pharmacological inhibitors of CaMKII.

**Figure 2:**
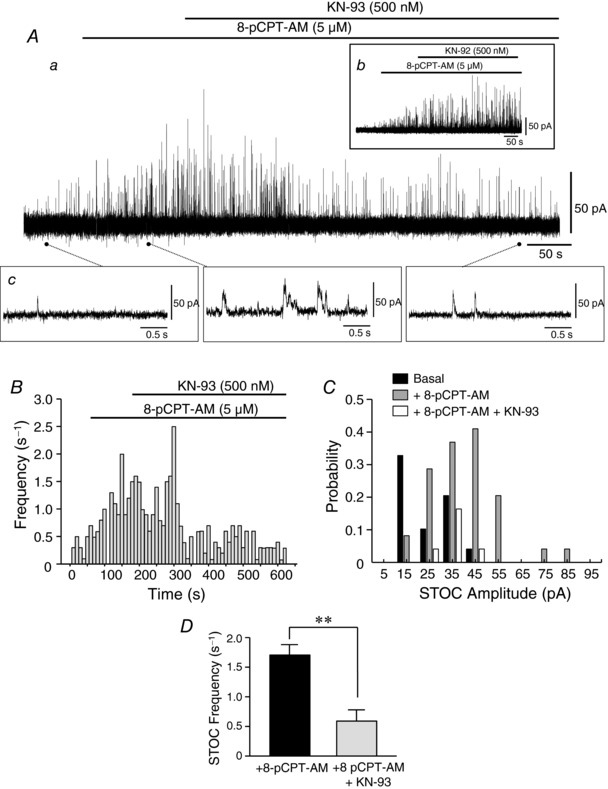
Epac‐mediated increases in STOC activity are reversed by KN‐93 inhibition of CaMKII in mesenteric smooth muscle cells *A*, STOCs recorded in isolated rat mesenteric smooth muscle cells using the whole‐cell recording technique. Upward deflections indicate the synchronized opening of groups of BK_Ca_ channels. Addition of 8‐pCPT‐AM (5 μm) to the superfusing bath solution at the point indicated by the bar above the trace increased both STOC frequency and amplitude. Examples of STOCs recorded in the presence and absence of 8‐pCPT‐AM are shown on an expanded timescale (*Ac*). STOCs were defined as events deviating from the baseline by a factor of 4 standard deviations above baseline noise. Application of the CaMKII inhibitor KN‐93 (500 nm) to the superfusing bath solution reversed the increase in STOC frequency and amplitude induced by 8‐pCPT‐AM (*Aa*, *P* < 0.05; *n* = 5). Application KN‐92 (500 nm) to the superfusing bath solution had no effect on STOC frequency and amplitude induced by 8‐pCPT‐AM (*Ab*; *n* = 3). *B*, histogram of STOC frequency against time for trace shown in (*A*). *C*, probability against STOC amplitude for trace shown in (*A*). *D*, summary of changes in STOC frequency induced by 8‐pCPT‐AM in experiments shown in (*A*) (mean ± SEM).

**Figure 3:**
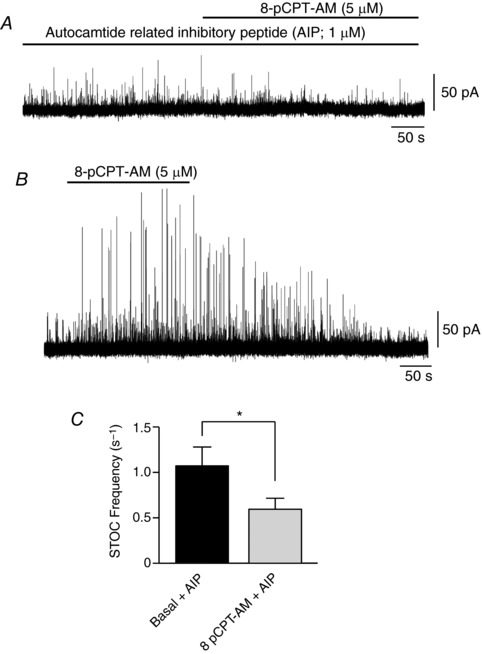
Autocamtide‐2‐inhibitory peptide blocks Epac‐mediated increases in STOC activity *A*, in whole‐cell recordings from isolated rat mesenteric smooth muscle cells, the ability of 8‐pCPT‐AM (5 μM) to reversibly increase STOC activity (*B*) is inhibited by inclusion in the pipette‐filling solution of autocamtide‐2‐inhibitory peptide (1 μm), a highly selective CaMKII inhibitor (*n* = 4). *C*, summary of STOC frequency following application of 8‐pCPT‐AM for the experiments shown in (*A*) (mean ± SEM).

In whole‐cell recordings from single, freshly isolated RMASMCs, application of the CaMKII inhibitor KN‐93 (500 nm) reversed the increase in STOC frequency (1.71 ± 0.22 to 0.61 ± 0.18 s^−1^; mean ± SEM) induced by 8‐pCPT‐AM (5 μm; *P* < 0.05; *n* = 5; paired *t* test) (Fig. 2). The increase in STOC amplitude was the result of an increase in the probability of larger events occurring in the presence of 8‐pCPT‐AM (Fig. 2 *C*). This was not mimicked by application of KN‐92 (500 nm), an inactive KN‐93 analogue (*n* = 3) (Fig. 2 *Ab*). Because KN‐93 has cellular effects other than inhibition of CaMKII (Pellicena & Schulman, 2014), we also undertook experiments using autocamtide‐2‐inhibitory peptide (AIP; 1 μm), a highly specific peptide inhibitor of CaMKII that corresponds to its autoinhibitory domain (Ishida *et al*. 1995). AIP was included in the pipette‐filling solution and allowed to dialyse into the cell for a minimum of 5 min on establishment of the whole‐cell configuration. In the presence of AIP, application of 8‐pCPT‐AM did not increase STOC frequency or amplitude in RMASMCs (Fig. 3 *A*; control response shown in Fig. 3 *B*). Indeed, there was a significant reduction in STOC frequency following 8‐pCPT‐AM addition from 1.08 ± 0.23 s^−1^ under basal conditions to 0.59 ± 0.12 s^−1^ in the presence of AIP (*P* < 0.05, *n* = 4; paired *t* test) (Fig. 3 *C*).

### Inhibition of inositol 1,4,5‐trisphosphate (IP_3_) receptors blocks Epac‐mediated CaMKII activation and increases in STOC activity

**Figure 4:**
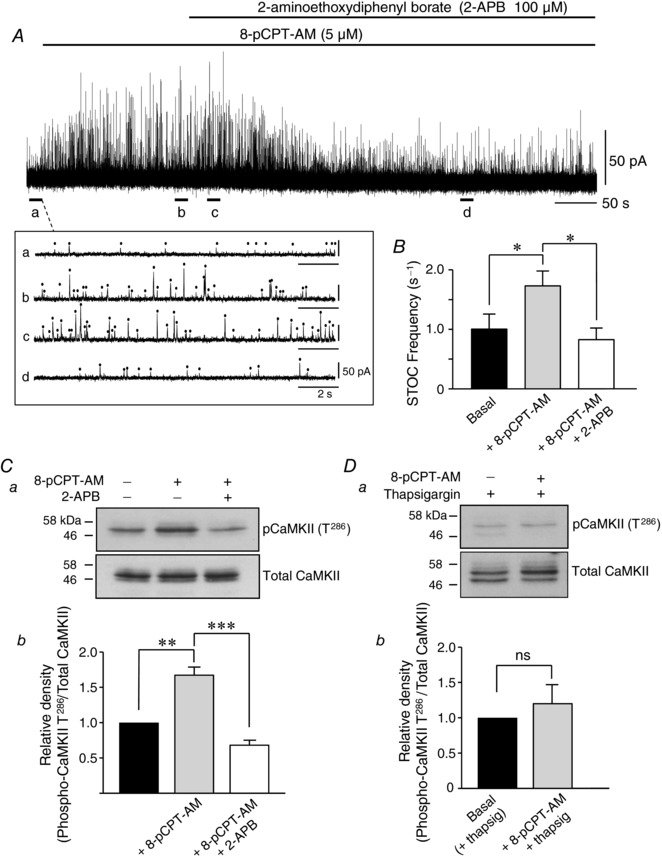
Inhibition of IP_3_ receptors blocks Epac‐mediated CaMKII phosphorylation and increases in STOC activity *A*, application of 2‐APB (100 μm), an IP_3_ receptor inhibitor, following STOC activation with 8‐pCPT‐AM caused an initial rapid increase in STOC frequency followed by a decline to levels significantly below those measured in 8‐pCPT‐AM alone (*P* < 0.05, *n* = 6). Whole‐cell recordings from isolated rat mesenteric smooth muscle cells held at −20 mV; expanded timescale of regions (*Aa*), (*Ab*), (*Ac*) and (*Ad*) shown below trace. *B*, summary of STOC frequency under basal conditions and following application of 8‐pCPT‐AM (5 μm) and 2‐APB (100 μm) (*n* = 6). *Ca*, Incubation of first‐order branches of rat mesenteric artery with 2‐APB (100 μm) significantly reduces the ability of 8‐pCPT‐AM to induce phosphorylation of specific CaMKII isoforms at Thr^286/7^ (pCaMKII (T^286/7^). Prior to homogenization, branches of rat mesenteric artery were incubated with vehicle control (DMSO) or 8‐pCPT‐AM (5 μm) for 5 min, or incubated with 8‐pCPT‐AM (5 μm) for 5 min following prior application of 2‐APB (100 μm) for 15 min. Proteins within the homogenates were separated on 10% polyacrylamide‐Tris gels and immunoblotted with an antibody directed against phospho‐CaMKII Thr^286/7^. Blot shown representative of three similar blots. *Cb*, densitometric analysis of experiment shown in (*Ca*). The relative density of the major phospho‐CaMKII T^286/7^ immunoreactive band (pCaMKII T^286/7^/total CaMKII) is shown normalized to basal levels (*n* = 3). *Da*, incubation of arteries with thapsigargin (500 nm) to deplete intracellular Ca^2+^ stores abolishes 8‐pCPT‐AM‐induced phosphorylation of CaMKII isoforms at Thr^286/7^. Blot shown representative of three similar blots. *Db*, densitometric analysis of experiment shown in (*Da*). The relative density of the major phospho‐CaMKII T^286/7^ immunoreactive band (pCaMKII T^286/7^/total CaMKII) is shown normalized to basal levels (*n* = 3).

Because CaMKII is activated by the binding of Ca^2+^/calmodulin (Soderling & Stull, 2001), we next explored possible sources of Ca^2+^ for Epac‐mediated CaMKII activation. 8‐pCPT‐AM was unable to increase STOC activity in RMASMCs pre‐incubated in 2‐aminoethoxydiphenyl borate (2‐APB) (100 μm), an IP_3_ receptor (IP_3_R) inhibitor. 2‐APB application following STOC activation with 8‐pCPT‐AM caused an initial rapid increase in STOC frequency followed by a decline to levels significantly below those measured in 8‐pCPT‐AM alone (from 1.72 ± 0.27 s^−1^ to 0.83 ± 0.23 s^−1^; *P* < 0.05; *n* = 6; Student–Newman–Keuls) (Fig. 4 *A* and *B*). These data suggest that Epac‐induced CaMKII activation may be triggered by Ca^2+^ release from intracellular stores via IP_3_Rs. To directly link the effects of 2‐APB to CaMKII activity, we stimulated rat mesenteric arteries with 8‐pCPT‐AM (5 μm) in the presence and absence of 2‐APB (100 μm) before homogenizing the tissue and quantifying the changes in CaMKII phosphorylation by immunoblot analysis using phospho‐specific antibodies against the CaMKII Thr286/7 autophosphorylation site. In the presence of 2‐APB, 8‐pCPT‐AM was unable to induce phosphorylation of Thr286/7, indicating that Epac‐induced Ca^2+^ efflux through IP_3_Rs is a probable mechanism that initiates CaMKII activity (*n* = 3) (Fig. 4 *C*). Consistent with the idea that store Ca^2+^ is needed to activate CaMKII, pretreatment with thapsigargin (500 nm), which depletes intracellular Ca^2+^ stores by blocking Ca^2+^ uptake via SERCA, also abolished 8‐pCPT‐AM‐induced phosphorylation of CaMKII at Thr286/7 (*n* = 3) (Fig. 4 *D*).

**Figure 5:**
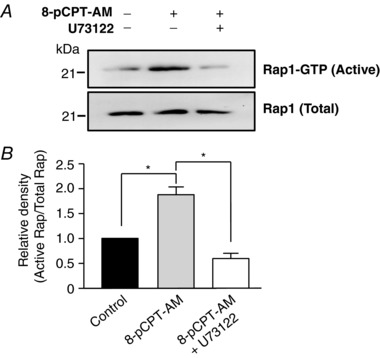
The PLC inhibitor U73122 blocks sustained Rap1 activation by 8‐pCPT‐AM *A*, exposure to 8‐pCPT‐AM increases the amount of activated (GTP‐bound) Rap1 (Rap1‐GTP) in proximal first‐order rat mesenteric arteries under control conditions (middle) but not following pre‐incubation with the PLC inhibitor U73122 (2 μm) (right; *n* = 3). Active GTP‐bound Rap1 levels were determined by incubating arterial lysates with GST‐RalGDS‐RBD fusion proteins followed by pull‐down on glutathione‐sepharose beads. Levels of captured Rap1‐GTP were quantified by western blot analysis. The total Rap1 level in these lysates was assessed by immunoblotting non‐pull‐down fractions with anti‐Rap1A/1B (Rap1 Total). *B*, densitometry analysis of Rap1 immunoblots (*n* = 3).

Upstream of IP_3_Rs, inhibition of the IP_3_‐generating enzyme phospholipase C (PLC) with U73122 (2 μm) significantly reduced the ability of 8‐pCPT‐AM to induce sustained activation of Rap1 (Fig. 5). Epac directly activates Rap1, which in turn can activate PLCε, an unusual PLC isoform that possesses an N‐terminal Ras GEF domain and two C‐terminal Ras binding domains (Kelley *et al*. 2001). Activated PLCε has two roles, further activation of Rap1 through GEF activity and phosphoinositol hydrolysis to produce IP_3_ (Oestreich *et al*. 2009). Active Rap levels were determined by incubating arterial lysates with fusion proteins comprising GST and the Rap‐binding domain of Ral‐guanine nucleotide‐dissociation stimulator (GST‐RalGDS‐RBD), which only bind the active GTP‐bound form of Rap1. Glutathione‐sepharose beads were then used to specifically pull‐down Rap1‐GTP, which was quantified by immunoblot analysis. The abolition of 8‐pCPT‐AM‐induced Rap activation by U73122 suggests that this PLC‐mediated feedback mechanism is essential for sustained Rap activity in vascular smooth muscle. Further investigation downstream of PLC using U73122 (i.e. its effect on Epac‐induced STOCs) was not undertaken as a result of the known side effect of U73122 on SR Ca^2+^ pumps (MacMillan & McCarron, 2010).

### 8‐pCPT‐AM does not directly activate BK_Ca_ channels

**Figure 6:**
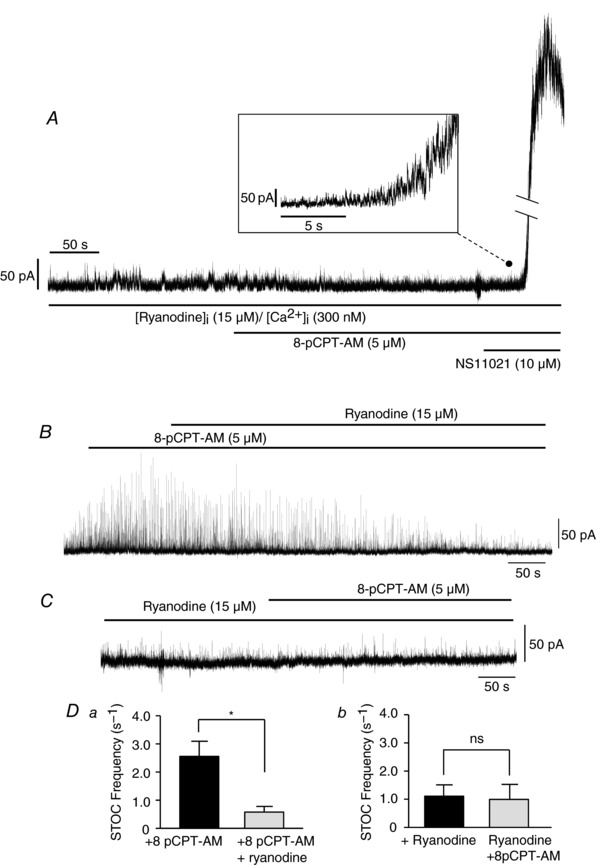
8‐pCPT‐AM does not directly activate BK_Ca_ channels but STOCs cannot be generated by 8‐pCPT‐AM in the presence of ryanodine *A*, whole‐cell recording of an isolated mesenteric myocyte held at −20 mV with ryanodine (15 μm) and Ca^2+^ (300 nm) included in the pipette‐filling solution. Ryanodine was allowed to dialyse into the cell for a minimum of 5–10 min on establishment of the whole‐cell configuration. Under these conditions RyR channels will be blocked but BK_Ca_ channels still active. Subsequent application of 8‐pCPT‐AM (5  μm) does not increase BK_Ca_ channel activity, but addition of the selective BK_Ca_ channel activator NS11021 (10  μm) induces a large outward current (*n* = 6). Break in current trace represents 1000 pA. *B*, STOCs recorded in isolated mesenteric myocytes using the whole‐cell recording technique. Application of ryanodine (15 μm) to the superfusing bath solution reversed the increases in STOC frequency and amplitude induced by the application of 8‐pCPT‐AM (5  μm; *n* = 4). *C*, in cells pre‐incubated in ryanodine (15 μm), application of 8‐pCPT‐AM does not increase STOC activity (*n* = 3). *Da*, summary of STOC frequency for experiments shown in (*B*) (mean ± SEM). *Db*, summary of STOC frequency for experiments shown in (*C*) (mean ± SEM).

We have previously shown that selective activation of Epac with 8‐pCPT‐AM in the presence of PKA inhibition increases the frequency of Ca^2+^ sparks (Roberts *et al*. 2013). In neurones, however, CaMKII phosphorylation of BK_Ca_ channels has been shown to increase their open probability by shifting their window of voltage activation towards more hyperpolarized membrane potentials (van Welie & du Lac, 2011). We therefore investigated whether a component of the 8‐pCPT‐AM effect on STOC activity could originate from direct effects on BK_Ca_ channels. In whole‐cell recordings from isolated mesenteric myocytes held at −20 mV with ryanodine (15 μm) and Ca^2+^ (300 nm) included in the pipette‐filling solution, application of 8‐pCPT‐AM (5  μm) failed to increase BK_Ca_ channel activity (*n* = 6) (Fig. 6 *A*). Under these conditions, Ca^2+^ release via RyRs will be blocked but BK_Ca_ channels will still be available for activation. This is demonstrated by the subsequent addition of the selective BK_Ca_ channel activator NS11021 (10 μm), which induced a large outward current (Fig. 6 *A*). Consistent with the idea that Epac‐mediated changes in STOC activity rely on underlying changes in the activity of RyRs, application of ryanodine (15 μm) to the superfusing bath solution reversed the increases in STOC activity induced by the application of 8‐pCPT‐AM (5  μm; *n* = 4) (Fig. 6 *B* and *Da*). Similarly, in cells pre‐incubated in ryanodine (15 μm), subsequent application of 8‐pCPT‐AM does not increase STOC activity (*n* = 3) (Fig. 6 *C* and *Db*).

### Epac activation decreases the rate of caffeine‐induced Ca^2+^ release from intracellular stores

**Figure 7:**
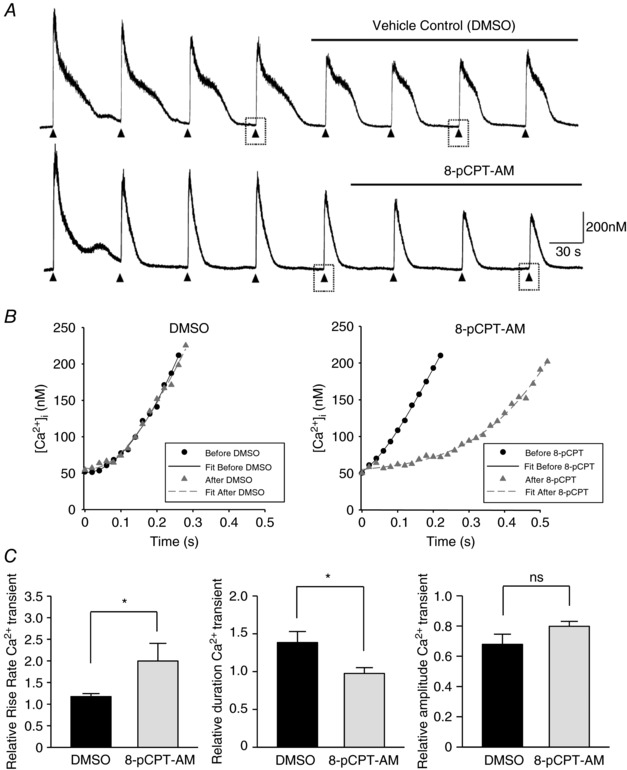
8‐pCPT‐AM slows the initial rate of [Ca^2+^]_i_ rise induced by caffeine without affecting the Ca^2+^ transient amplitude *A*, train of Ca^2+^ transients triggered by pulse application of 5 mm caffeine; point of application indicated by an arrowhead. Extracellular solution was switched to test solution containing DMSO (upper) or 5 μm 8‐pCPT‐AM (lower) at the point indicated by bar above trace. *B*, initial Ca^2+^ rise rate for transients before and after the application of vehicle control (DMSO; left) or 5 μm 8‐pCPT‐AM (right) was fitted with a third‐order polynomial equation. In each case, the rise rate of the third Ca^2+^ transient after switching to the test solution was normalized against that of the Ca^2+^ transient immediately before solution change (analysed transients indicated by dashed box). *C*, summary of Ca^2+^ transient parameters expressed as the mean ± SEM for DMSO‐treated and 8‐pCPT‐AM treated cells (*n* = 5 and 4, respectively).

Epac activation may increase RyR activity by enhancing RyR Ca^2+^ sensitivity, or by increasing the Ca^2+^ content of the RyR‐accessible Ca^2+^ store. We thus measured the effect of Epac activation on store content and the rate of Ca^2+^ clearance from the cytosol by inducing transient Ca^2+^ release from the SR by pulse applications of the RyR activator caffeine in voltage clamped and fura‐2‐dialysed myocytes (Fig. 7).

Repeated 100 ms application of caffeine (5 mm; indicated by arrow heads) at 60 s intervals triggered a train of Ca^2+^ release transients (Fig. 7 *A*). At the point indicated by the bar above the trace, the extracellular superfusing solution was switched to test solutions containing either vehicle control (DMSO) or 8‐pCPT‐AM (5 μm). In both cases, the amplitude of the caffeine‐induced Ca^2+^ transients decreased during the course of the experiment, suggesting that the SR Ca^2+^ is being slowly depleted. Epac activation with 8‐pCPT‐AM had no significant effect on Ca^2+^ transient amplitude during the course of the experiment (relative amplitude for DMSO‐treated cells 0.68 ± 0.07 and for 8‐pCPT‐AM treated cells 0.80 ± 0.03, *n* = 5, 4 respectively; *P* > 0.05; unpaired *t* test) (Fig. 7 *C*, right). There was however a significant shortening of the relative duration of the transient following Epac activation (DMSO treated cells 1.39 ± 0.15 and 8‐pCPT‐AM treated cells 0.98 ± 0.08, *n* = 5, 4 respectively; *P* < 0.05; unpaired *t* test) (Fig. 7 *C*, middle).

During the experiments, it was noted that the initial upward deflection of the Ca^2+^ transient appeared slower following 8‐pCPT‐AM application. Thus, the early period of [Ca^2+^]_i_ increase in response to caffeine was examined more closely. The initial rate of Ca^2+^ rise (from baseline to [Ca^2+^]_i_ of 200 nm) was almost indistinguishable before and after DMSO application, despite the later Ca^2+^ transients being smaller in amplitude (Fig. 7 *B*, left). The initial rate of Ca^2+^ rise was, however, considerably slower after application of 8‐pCPT‐AM (5 μm) (Fig. 7 *B*, right). The relative initial rate of Ca^2+^ rise was 1.17 ± 0.07 and 2.00 ± 0.41 for DMSO and 8‐pCPT‐AM treated cells respectively (*n* = 4, 5; *P* < 0.05, unpaired *t* test) (Fig. 7 *C*, left). These results suggest that Epac activation has no significant effect upon store content, although it may affect the kinetic behaviour of the RyR release channels.

**Figure 8:**
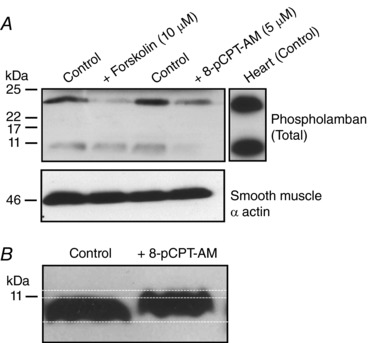
8‐pCPT‐AM exposure induces changes in phospholamban solubility and gel mobility *A*, first‐order branches of rat mesenteric artery were incubated in vehicle control (DMSO), the adenylyl cyclase activator, forskolin (10 μm) or 8‐pCPT‐AM (5 μm). Arteries were subsequently homogenized in lysis buffer containing 0.5% Triton‐X for 10 min on ice and homogenates spun at 16 000 g for 10 min to pellet insoluble fractions. Proteins within the supernatants were separated by SDS‐PAGE, transferred electrophoretically onto nitrocellulose membrane and immunoblotted with antibodies against phospholamban. Untreated rat heart lysates were run as a control (right). The two bands represent the phospholamban monomer (6 kDa) and the phospholamban pentamer (24 kDa). Treatment with forskolin or 8‐pCPT‐AM reduces the amount of soluble phospholamban. Membranes were stripped and re‐probed with anti‐smooth muscle actin as a loading control (lower). *B*, treatment with 8‐pCPT‐AM causes the phospholamban monomer to run at a higher molecular weight in 15% Tris gels.

Epac and subsequent CaMKII activation probably induces changes in RyR activity through phosphorylation of the release channel itself or associated regulatory proteins. We were unable to detect 8‐pCPT‐AM‐induced phosphorylation of RyR at canonical CaMKII phosphorylation sites. This may simply reflect the levels of these proteins within vascular smooth muscle cells, which is considerably less than in cardiomyocytes. Equally, we could not reliably detect phosphorylation of the SERCA pump regulator phospholamban at Thr^17^, another key CaMKII target in the heart. Interestingly, we did see 8‐pCPT‐AM‐mediated changes with respect to both phospholamban mobility within gels and susceptibility to detergent extraction from membranes, which may indicate protein modification following Epac activation (Fig. 8).

## Discussion

The Ca^2+^‐sensing enzyme CaMKII is a well‐established and important modulator of cardiomyocyte contraction, although its role in the control of vascular tone is surprisingly poorly understood (Toussaint *et al*. 2016). In the heart, aside from its Ca^2+^ sensitivity, CaMKII responds to the cellular redox state through oxidation of methionine residues in its regulatory domain (Erickson *et al*. 2008). However, in differentiated vascular smooth muscle, which relies on complex patterns of Ca^2+^ signalling and functions to translate changes in oxidative stress into contractile behaviour, little is known about its role. We show that Epac‐mediated activation of membrane currents driving smooth muscle hyperpolarization occur via the activation of CaMKII. To our knowledge this is the first report of CaMKII activation being associated with the initiation of vasorelaxation.

Activation of Epac has previously been shown to induce smooth muscle relaxation by increasing the frequency of localized Ca^2+^ release from RyRs located on the peripheral sarcoplasmic reticulum (Roberts *et al*. 2013). These subsurface Ca^2+^ sparks activate BK_Ca_ channels in the plasma membrane, evoking STOCs that hyperpolarize the cell and reduce voltage‐dependent Ca^2+^ entry (Roberts *et al*. 2013). In the present study, we investigate the mechanism by which Epac increases STOC activity. We show that (i) the ability of Epac to increase STOC frequency and amplitude in rat mesenteric artery smooth muscle cells depends upon the activation of CaMKII; (ii) Epac activation preferentially induces autophosphorylation of specific CaMKII γ isoform/s; (iii) Epac‐induced CaMKII activation is probably initiated by IP_3_‐mobilized Ca^2+^; and (iv) Epac activation has little effect on intracellular Ca^2+^ store content but affects caffeine‐induced store release, possibly as a result of changes in the kinetic behaviour or synchronous opening of RyRs. We do not consider that the observed increase in STOC activity is attributable to Epac‐activated CaMKII directly phosphorylating and activating BK_Ca_ channels. We see no significant BK_Ca_ channel activation by 8‐pCPT‐AM under conditions where RyR channels are blocked, even though BK_Ca_ channels are still susceptible to activation by NS11021.

The main CaMKII holoenzymes found in vascular smooth muscle form from splice products from γ and δ genes (Singer, 2012). Consistent with this, we find messenger RNA encoding at least two CaMKII γ variants and two CaMKII δ variants in rat mesenteric artery. The expression of multiple CaMKII isoforms in this tissue is confirmed by immunoblots of arterial lysates using pan‐specific CaMKII antibodies that produce three/four immunoreactive bands, only one of which is significantly phosphorylated following Epac activation. By immunoblotting arterial tissue obtained from mice in which the gene encoding either CaMKIIδ or CaMKIIγ had been globally deleted (Backs *et al*. 2009, 2010), we were able to determine that Epac activation predominantly induces phosphorylation of CaMKII γ variants. This is consistent with the finding that CaMKIIγ expression dominates in differentiated smooth muscle (Kim *et al*. 2000; Marganski *et al*. 2005). CaMKIIδ, in contrast, is a relatively minor isoform in contractile muscle. It is up‐regulated in synthetic/proliferating smooth muscle and is required for injury‐induced neointima formation, largely through its regulation of the expression and activity of cell cycle activators (Li *et al*. 2011). We cannot entirely rule out the possibility that δ variants make up a minor component of the immunoreactive band that strongly binds phospho‐CaMKII antibodies following 8‐pCPT‐AM treatment. Faint anti‐CaMKII immunoreactive bands persist at this molecular weight in CaMKIIγ knockout lysates, and it is possible that δ and γ subunits exist together in the functional holoenzyme complex. Interestingly, the two major PCR products we detect using primers designed to amplify γ isoforms are predicted splice variants of the γ gene whose expression has never been reported. Thus, the CaMKII isoforms involved in this Epac‐activated pathway may be novel. We did not undertake any functional work with the δ or γ knockout tissue because CaMKIIδ and CaMKIIγ show redundancy and are able to functionally compensate for each other (Kreusser *et al*. 2014).

Because these experiments were conducted in whole arteries, we cannot fully distinguish whether the isoforms we detect are in smooth muscle cells, endothelial cells or innervating nerves. However, our electrophysiological experiments have all been conducted in single isolated smooth muscle cells and clearly show that inhibition of CaMKII activity by KN‐93 or AIP, a highly specific peptide inhibitor of CaMKII, blocks the ability of Epac to generate STOC activity.

Intuitively, as a Ca^2+^‐sensing enzyme, agents that elevate intracellular Ca^2+^ should activate CaMKII and, indeed, in large blood vessels, CaMKII is activated in response to contractile stimuli and this activation maintained throughout tonic contraction (Kim *et al*. 2000; Rokolya & Singer, 2000). In ferret aortae, inhibition of CaMKII with KN‐93 decreases the amplitude of contraction induced by high extracellular K^+^, although it has no significant effect on the size of contractions induced by the α adrenoceptor agonist phenylephrine (Kim *et al*. 2000). Similarly, in pig carotid artery, KN‐93 inhibits both the amplitude of contraction and the ability to maintain force in response to high extracellular K^+^, although it inhibits only force maintenance in response to histamine (Rokolya & Singer, 2000). It is suggested that the site of Ca^2+^ release/influx and/or calmodulin compartmentalization controls the spatial activation of CaMKII in response to different upstream stimuli. This would be in keeping with studies in cardiomyocytes that demonstrate distinct pools of CaMKIIδ linked to different upstream pathways (Mishra *et al*. 2011). This specificity of activation is controlled by the mobilization of different Ca^2+^ stores as opposed to the subcellular compartmentation of different subtypes of the enzyme.

By contrast to large vessels, in mesenteric arteries from transgenic mice expressing the inhibitor peptide CaMKIIN in smooth muscle, CaMKII inhibition has no effect on vasoconstriction in response to KCl, angiotensin II, or phenylephrine (Prasad *et al*., 2013). Instead, CaMKII supports intracellular Ca^2+^ homeostasis by phosphorylating and maintaining the activity of voltage‐dependent Ca^2+^ channels and phospholamban. The functionality of Epac‐induced CaMKII activation in mediating vasorelaxation will presumably rely on the selective activation of CaMKII holoenzymes residing sufficiently close to activating IP_3_‐sensitive release channels and downstream targets such as RyRs and their associated regulatory proteins. We would predict that these pools of CaMKII are distinctly accessible to Epac‐mediated upstream events and shielded from contractile stimuli.

Our data suggest that CaMKII activity in vascular smooth muscle is triggered by Epac‐induced Ca^2+^ efflux through IP_3_Rs. The immediate downstream target of Epac is the small‐Ras‐related G‐protein Rap1. We propose that, following activation, Rap interacts with the C‐terminal Ras binding domains of PLCε (Kelley *et al*. 2001). Subsequent activation of PLCε results in two separate events: phosphoinositol hydrolysis and the formation of IP_3_ and further activation of Rap through the N‐terminal Ras GEF domain of PLCε. Consistent with this model, the PLC inhibitor U73122 significantly reduced the ability of the selective Epac activator, 8‐pCPT‐AM, to induce sustained activation of Rap1. Indeed, the abolition of 8‐pCPT‐AM‐induced Rap activation by U73122 suggests that this PLC‐mediated feedback mechanism is essential for sustained Rap activation in vascular smooth muscle. We also find that, in the presence of the IP_3_ receptor inhibitor 2‐APB, 8‐pCPT‐AM is unable to induce phosphorylation of CaMKII at Thr286/7 or increase STOC frequency/amplitude, which would be consistent with IP_3_‐released Ca^2+^ being essential for activation of the CaMKII holoenzyme. Pretreatment with thapsigargin, which depletes intracellular Ca^2+^ stores by blocking Ca^2+^ uptake via SERCA, also abolished 8‐pCPT‐AM‐induced CaMKII phosphorylation. During the course of the experiments, we noted that 2‐APB application following STOC activation with 8‐pCPT‐AM had a bi‐phasic effect on STOC activity, causing an initial rapid increase in STOC frequency followed by a decline to levels significantly below those measured in 8‐pCPT‐AM alone. Further experiments showed that application of 2‐APB alone caused a transient increase in basal STOC frequency (data not shown), which may indicate a constant Ca^2+^ leak from a common Ca^2+^ store via IP_3_Rs which, when blocked, alters store content and RyR activity. We see some evidence of basal phosphorylation of CaMKII in immunoblots, which may be consistent with this idea.

Downstream of CaMKII activation, we investigated possible mechanisms by which CaMKII could affect STOC activity. STOCs are generated by the synchronized opening of groups of sarcolemmal BK_Ca_ channels in response to localized Ca^2+^ release from RyRs on the subjacent SR. Changes in STOC frequency or amplitude thus give a direct indication of changes in underlying behaviour of RyRs. To increase STOC activity, Epac may affect RyR behaviour directly via enhancement of Ca^2+^ sensitivity, or indirectly by increasing the content of the RyR‐accessible Ca^2+^ store. We thus measured the effect of Epac activation on store content and the rate of Ca^2+^ clearance from the cytosol by inducing transient Ca^2+^ release from the SR by pulse applications of the RyR activator caffeine in voltage clamped and fura‐2‐dialysed myocytes. Epac activation significantly slowed the initial global rise in [Ca^2+^]_i_ in response to caffeine but had no effect on the relative transient amplitude. This suggests that depleted SR Ca^2+^ content is probably not the reason for the sluggish Ca^2+^ rise in 8‐pCPT‐AM treated cells. A possible explanation for these observations is that the rapid and synchronized opening of RyRs is compromised by Epac activation. CaMKII probably induces changes in RyR activity through phosphorylation of the release channel itself or associated regulatory proteins (Van Petegem, 2012). RyR1, 2 and 3 have been reported to be expressed in vascular smooth muscle and all contain potential phosphorylation sites for CaMKII. We were unable to detect 8‐pCPT‐AM‐induced phosphorylation of vascular RyRs using phospho‐specific antibodies against serine 2814, the CaMKII phosphorylation sites on RyR2 that enhances Ca^2+^ sensitivity and increases RyR open probability in cardiac muscle (Ai *et al*. 2005). This may simply reflect the low level of RyR proteins within vascular smooth muscle or phosphorylation at an alternative site. CaMKII may also directly or indirectly modify the behaviour of associated regulatory proteins such as FK‐506 binding protein, which functions to synchronize the activity of RyRs by coupling the opening and closure of neighbouring channels (Marx *et al*. 1998). Loss of the ability to simultaneously open in response to caffeine would account for our Epac‐mediated slowing of global [Ca^2+^]_i_ rise. Although we see no clear changes in SR content following activation of Epac, we cannot rule out an increase in uptake that balances the increased store leak (spark activity). As with RyR, we could not reliably detect phosphorylation of the SERCA pump regulator phospholamban at Thr^17^, another key CaMKII target in the heart. Interestingly, we did see 8‐pCPT‐AM‐mediated changes in both phospholamban mobility within gels and susceptibility to detergent extraction from membranes, which may indicate protein modification following Epac activation (Bidlack & Shamoo, 1980).

In conclusion, the results of the present study indicate that Epac‐induced STOC activity in contractile vascular smooth muscle occurs via the activation of CaMKII and suggests that functionally distinct pools of CaMKII may be pivotal in mediating cyclic AMP‐induced vasodilatation.

## Additional information

### Competing interests

The authors declare that they have no competing interests.

### Author contributions

ESAH, TK, JMQ and CD were responsible for the conception and design of experiments. ESAH was responsible for the electrophysiological, biochemical and molecular experiments. TK was responsible for the calcium fluorometry experiments. ESAH, TK and CD were responsible for analysis and interpretation of data. ESAH and CD were responsible for drafting the article. ESAH, TK, JMQ and CD were responsible for revising the article critically for important intellectual content and approving the final version to be published.

### Funding

This work is supported by a British Heart Foundation project grant PG/14/55/30973 and BBSRC Doctoral Training Grant (DTP1‐NLD) to ESAH.
